# CHRONIC URTICARIA AND TREATMENT OPTIONS

**DOI:** 10.4103/0019-5154.57603

**Published:** 2009

**Authors:** Kiran Vasant Godse

**Affiliations:** *From Shree Skin Centre, 22, L Market, Sector 8, Nerul, Navi Mumbai - 400 706, India.*

**Keywords:** *Urticaria*, *autoimmune urticaria*, *autoantibodies*

## Abstract

Chronic urticaria has a wide spectrum of clinical presentations and causes. Still, despite our best efforts no cause may be found in the majority of cases. The treatment options are: Primary prevention in the form of avoidance of aggravating factors; counseling; antihistamines; leukotriene receptor antagonists; prednisolone; sulfasalazine and a host of immunosuppressives like methotrexate, cyclosporine, omalizumab etc.

## Introduction

Chronic urticaria (CU) has a wide spectrum of clinical presentations and causes. An affected patient often goes from one dermatologist to another in pursuit of a cure. Despite a dermatologist's best efforts no cause can be found in most cases. About 30-50% of patients with chronic idiopathic urticaria (CIU) have circulating histamine releasing autoantibodies to the high-affinity IgE receptor FcɛRI on basophils and mast cells or, less commonly, antibodies to IgE. The term autoimmune urticaria is increasingly being accepted for this subgroup of patients.[1] Urticaria has wide presentations. Wheals may occur almost daily in some individuals or it may occur periodically in others. It may present as relapse after remission of urticaria few to several weeks later. The morphology of wheals often gives clue to physical urticarias. Angioedema is often associated with CU. Deep dermal swellings of delayed pressure urticaria may create confusion in the mind of the clinician when it comes to differentiating with angioedema.

In many patients, in spite of extensive investigations, the cause remains elusive. The term idiopathic is often used to denote this category. Now it is known that autoimmunity is the cause of CU in 50% of cases. Allergy is probably not the cause of CU. Only thyroid autoimmunity is often associated with the condition.[2] Dietary pseudoallergens may aggravate existing CU analogous perhaps to the adverse effect of nonsteroidal anti-inflammatory drugs (NSAIDs).[3]

Infection as a cause of CU is based on uncontrolled series of reports of patients with dental abscesses. Intestinal parasitosis as a cause of CU is rare in developed countries. One study from newly developed city of Navi Mumbai had similar findings.[4] Another study found patients with delayed pressure urticaria and those with positive autologous serum skin test (ASST) reported serious impairment of quality of life in Indian patients.[5]

## Aggravating Factors

There are number of aggravating factors that can be avoided by simple measures. The treating physician can identify the same with careful history taking. These include diet, drugs, alcohol, viral infections, local heat and friction, and mental stress.[3]

In India, diet is often considered as a cause of any skin allergy and often patients come to the physician with a list of ‘not to eat’ things. Now we know that pseudoallergens may be important cause in some patients.[6] Pseudoallergic reactions to additives, natural salicylates, and aromatic compounds are almost certainly dose related. We do not know how much is to be ingested to precipitate an attack. In one study, only 19% of patients reacted severely to challenge capsules containing food additives and salicylic acid.[6]

Aspirin is the commonest drug to aggravate urticaria. Aspirin and other NSAIDs can worsen CU in 20-30% of patients during active phase but probably not in remission.[7]

Overheating and local pressure of belts and clothing aggravate CU and there is often an overlap between physical urticaria and CU.

Alcohol can worsen urticaria by the mechanism of vasodilatation. Upregulation of cytokines with the acute phase response, leading to temporary state of enhanced mast cell releasability is the probable mechanism for aggaravation of urticaria during viral infections.[3]

## Treatment

A clear explanation that CU is not allergic is important to address since inevitable conviction many patients hold that diet is a cause. Important information to patients must include useful websites and written information about the disease. Treatment plan should include treatment of identifiable cause, avoidance of aggravating factors, advice and written information about the condition, and antihistamines trial. Topical lotions in form of calamine lotion, menthol with aqueous cream, and crotamiton lotion are useful soothing agents in the treatment.

### Antihistamines

They are the first line treatment for all patients with CU. Classic H1 antihistamines with sedation as a side effect include chlorpheniramine, hydroxyzine, and diphenhydramine. Nonsedating second generation H1 antihistamines include loratadine, cetirizine, terfenadine, and mizolastine. Second generation H1 antihistamine derivatives include desloratadine, levocetirizine, and fexofenadine. H2 antihistamines include cimetidine, ranitidine, famotidine and nizatadine.

Treatment is generally started with nonsedating antihistamine in the daytime and sedating antihistamine in the night. In the licenced dosage all antihistamines are equal in efficacy and there is little to chose between the different molecules. It is common to double or triple the dosage of nonsedating antihistamines if patients do not respond to standard dosage. H2 antihistamines can be added if patients complain of indigestion or acidity. Combination often helps the patient. A study found fexofenadine better than generic levocetirizine in a small trial.[8] Many patients do not respond to these combinations and second-line therapies come into picture. One study from secondary referral center found that 40% patients did not respond to antihistamines.[9]

### Second line treatments

Doxepin is the second line of treatment if antihistamines do not work. It is a tricyclic antidepressant. Dosage is 10-25 mg and may be increased to 50 mg. It works well when taken at night. Greene *et al*., showed its superiority over diphenhydramine.[10] Its main side effects are drowsiness, dry mouth, metallic taste, constipation, urinary retention, blurred vision, palpitation, and tachycardia.

Montelukast is a leukotriene receptor antagonist. It does not work in all types of urticaria. It works well for aspirin-sensitive urticaria.[11] A study found montelukast monotherapy ineffective in CIU when compared with cetirizine.[12] Daily dosage is 10 mg taken at bedtime. There are no important drug interactions.

Prednisolone has predominant glucocorticoid activity and is commonly used for long-term disease suppression. There are few studies on prednisolone and urticaria. They should be used cautiously and for only short periods due to the side effects. Prednisolone in the dose of 20-30 mg for three days is effective to control severe attacks of urticaria and angioedema. Low dosage alternate day dosing may be used in patients with CU who do not respond to other therapies.[13] Systemic corticosteroids act at multiple levels, they suppress the formation of antibodies, they have a strong anti-inflammatory effect, and in addition, are known to decrease the production of histamine-releasing factors.[14] Steroids should be used with care in patients with diabetes, hypertension, and osteoporosis.

Sulfasalazine is a long acting sulfonamide used in the treatment of ulcerative colitis, rheumatoid arthritis, and Crohn's disease. It is reported to be useful in steroid-dependent CU.[15] Starting dose is 1 gm twice daily. Blood count and liver and renal function tests should be checked monthly for period of three months and every three months. Side effects include anemia, rashes, exfoliative dermatitis, neuropathy, and depression.

### Third line treatments

Methotrexate is the right choice in Indian setting where cost is an important factor in deciding the therapy. In India, methotrexate has the potential of being a viable option for the treatment of resistant autoimmune urticaria (AIU) as it is cost effective and most dermatologists have the experience of using it for psoriasis. Methotrexate is a derivative of folic acid that interferes with dihydrofolate reductase and the production of DNA in actively dividing cells. A study found methotrexate effective in patients with autoimmune urticaria in a dose of 2.5 mg orally twice a day on Saturday and Sunday of every week. Informed consent was obtained before starting methotrexate. In addition, cetirizine 10 mg and folic acid 1.5 mg were given daily. All four patients showed a remarkable remission in the form of reduction in whealing and itching in one month.[16] Most important side effects are bone marrow suppression and hepatitis.

Cyclosporine is a powerful inhibitor of both cell mediated and humoral responses. It inhibits the release of histamine from basophils and tumor necrosis factor α production by mast cells. There are many reports of efficacy of cyclosporine in urticaria.[17,18]

Dosage of cyclosporine varies from 3-5 mg/kg as a starting dose and reduced over three to four months. One study showed that prolonged treatment with cyclosporine is beneficial for maintaining remission in severe cases of CU. It spares the need for corticosteroids and is accompanied with mild side effects.[19] The main contraindications are impaired kidney function, uncontrolled blood pressure, active serious infections, and cancers.

Omalizumab, a recombinant humanized monoclonal antibody against immunoglobulin IgE, represents a unique therapeutic approach for the treatment of allergic diseases. This agent acts as a neutralizing antibody by binding IgE at the same site as the high-affinity receptor. Subsequently, IgE is prevented from sensitizing cells bearing high-affinity receptors. Inhibition of the biological effects of IgE targets an early phase of the allergic cascade before the generation of allergic symptoms.[20] There are reports of efficacy of omalizumab in CU.[21] One Indian study found this molecule effective in a patient where cyclosporine failed to show response.[22]

Bajaj *et al*., have used autologous serum therapy (AST) for two groups of patients with CIU (ASST-positive and ASST-negative) and found it to be an effective therapeutic modality to reduce disease severity as well as antihistamine requirement. This also prevented relapse of symptoms for durations as long as two years.[23]

Intravenous immunoglobulin (0.4 g/kg for 5 days) was effective in relieving symptoms in patients with chronic AIU and achieving ASST negativity as well as long-term (>3 years) remission. Complete, permanent remission was reported in patients who attained ASST negativity within six months of therapy.[24] Although the exact mechanism of action is unknown, presence of anti-idiotypic antibodies capable of suppressing IgE autoantibodies, in the intravenous immunoglobulin (IVIG) preparation has been suggested.[25]

Plasmapheresis was found to be beneficial in a small series of patients with AIU by eliminating the functional autoantibodies from system.[26]

Mycophenolate mofetil may be a valuable and safe treatment for patients with CU who do not respond to antihistamines and/or corticosteroids, and who require aggressive treatment to control their disease symptoms.[27]

Most of the above therapies are costly and cannot be used routinely in an Indian setting.

In summary, methotrexate and autologous serum therapy are viable options in CU not responding to first and second line of treatments.
